# Impact of air pollution on hospital admissions with a focus on respiratory diseases: a time-series multi-city analysis

**DOI:** 10.1007/s11356-019-04781-3

**Published:** 2019-03-30

**Authors:** Alessandro Slama, Andrzej Śliwczyński, Jolanta Woźnica, Maciej Zdrolik, Bartłomiej Wiśnicki, Jakub Kubajek, Olga Turżańska-Wieczorek, Dariusz Gozdowski, Waldemar Wierzba, Edward Franek

**Affiliations:** 1 Central Clinical Hospital MSWiA in Warsaw, Wołoska 137, 02-507 Warsaw, Poland; 2 University of Humanities and Economics in Łodz, Satellite Campus in Warsaw, ul. Wolność 2a, 01-018 Warsaw, Poland; 3 Chancellery of the Prime Minister of Poland, al. Ujazdowskie 1/3, 00-001 Warsaw, Poland; 4 Warsaw University of Life Sciences, Nowoursynowska 166, 02-787 Warsaw, Poland; 5 Mossakowski Clinical Research Centre, Polish Academy of Sciences, Pawinskiego 5, 02-106 Warsaw, Poland

**Keywords:** Air pollution, Respiratory health, Hospital admissions, Multi-city time-series analysis, Particulate matter

## Abstract

Together with the growing availability of data from electronic records from healthcare providers and healthcare systems, an assessment of associations between different environmental parameters (e.g., pollution levels and meteorological data) and hospitalizations, morbidity, and mortality has become possible. This study aimed to assess the association of air pollution and hospitalizations using a large database comprising almost all hospitalizations in Poland. This time-series analysis has been conducted in five cities in Poland (Warsaw, Białystok, Bielsko-Biała, Kraków, Gdańsk) over a period of almost 4 years (2014–2017, 1255 days), covering more than 20 million of hospitalizations. The hospitalizations have been extracted from the National Health Fund registries as daily summaries. Correlation analysis and distributed lag nonlinear models have been used to investigate for statistically relevant associations of air pollutants on hospitalizations, trying by various methods to minimize potential bias from atmospheric parameters, days of the week, bank holidays, etc. A statistically significant increase of respiratory disease hospitalizations has been detected after peaks of particulate matter concentrations (particularly PM_2.5_, between 0.9 and 4.5% increase per 10 units of pollutant increase, and PM_10_, between 0.9 and 3.5% per 10 units of pollutant increase), with a typical time lag between the pollutant peak and the event of 2 to 6 days. For other pollution parameters and other types of hospitalizations (e.g., cardiovascular events, eye and skin diseases, etc.), a weaker and ununiform correlations were recorded. Ambient air pollution exposure increases are associated with a short-term increase of hospitalizations due to respiratory tract diseases. The most prominent effect was recorded with the correlation of PM_2.5_ and PM_10_. There is only weak evidence indicating that such short-term associations exist between peaks of air pollution concentrations and increased hospitalizations for other (e.g., cardiovascular) diseases. The obtained information could be used to better predict hospitalization patterns and costs for the healthcare system and perhaps trigger additional vigilance on particulate matter pollution in the cities.

## Introduction

Ambient air pollution is recognized to adversely affect health (Arbex et al. 2012). Several studies conducted in almost all parts of the world have found that day-to-day increases in pollution levels are associated with different pathologies, respiratory tract disease (Kim et al. 2018; Cerezo Hernández et al. 2018), asthma (Zheng et al. 2015), increased COPD exacerbations (Moore et al. 2016), cardiovascular (Analitis et al. 2006) and cerebrovascular diseases—stroke (Tian et al. 2017), etc. Several theories on the pathogenesis of these effects of ambient pollution have been put forward (Bernstein et al. 2004), but overall, the area remains poorly understood, and there is no consensus on which constituents (Kampa and Castanas 2008) of air pollution are most harmful (Brunekreef and Holgate 2002). The large Polish (Nabrdalic and Samora 2018) cities and in general Eastern European cities are recognized to have poorer air quality relative to other cities in Europe (Katsouyanni et al. 1996; Zmirou et al. 1998). Higher pollution levels in Polish cities are caused, in part, by sources that include coal-powered electricity generating stations and heating sources. Despite the recognition that Polish cities have perhaps poorer air quality than other Western European cities, up to date only few large-scale statistical analysis have been performed (Pac et al. 2013; Haluszka et al. 1998; Niepsuj et al. 1998) on the potential impact associations between day-to-day fluctuations in air pollution levels and hospitalizations. At this time, we know of no study that analyses data from more than 20 million hospitalizations and ED visits (ED visits which turn into hospitalizations and/or ED visits which require a hospital diagnostic or specialized medical visit or intervention are logged into the database with an ICD-10 classification and therefore will be reported as “hospitalizations”—these data will include the specialized ambulatory care) in the study area. The goal of this analysis was to investigate the association between different air pollutants and hospitalizations in a multi-city time-series observation, considering the potential influence of key meteorological parameters. This data analysis has been possible thanks to the access to the electronic registry of the Polish National Healthcare fund, to the data of the Institute of Meteorology and Water Management and Chief Inspectorate of Environmental Policy.

## Methods

### Core data source

The data related to the number of hospitalizations in the cities of Warsaw, Białystok, Bielsko-Biała, Kraków, and Gdańsk were obtained from the reporting system of the NHF (in Polish: Narodowy Fundusz Zdrowia) and covered a period of almost 4 years (2014–2017, 1255 days). The International Classification of Diseases 10th (ICD-10) revision coding was used to identify the different diagnoses at admission (the following ICD-10 categories were considered: F, mental and behavioral disorders; G, diseases of the nervous system; H, dis. of the eye, adnexa, of the ear mastoid process; I, diseases of the circulatory system; J, diseases of the respiratory system; L, diseases of the skin and subcutaneous tissue; S/T, injury, poisoning, and other cons. of ext. causes) for the period between January 1, 2014, and August 1, 2017.

Data on the concentration of air pollution were obtained from the Chief Inspectorate for Environmental Protection (GIOS) and included NO, NO_*x*_, NO_2_, O_3_, SO_2_, PM_2.5_, PM_10_, PM_10__24, and PM_2.5__24. Daily (obtained from manual stations) and hourly data (obtained from automatic stations, coded as 24) have been used in the analysis. Meteorological data have been gathered from the Institute of Meteorology and Water Management (IMGW) that have beacons in the Polish cities and included temperature, main wind speed, and precipitations.

### Sample preparation

In this time-series analysis, to account for the great data variability encountered on the different week days (Faryar 2013; De Pablo Dávila et al. 2013; Sun et al. 2009; Tai et al. 2006), we normalized the data sample per week day, season, and bank holidays, calculating a ratio of observed number of patients by mean number of patients in the particular day of the week, or holiday. In addition, and specifically for the analysis explained in the “Cardiovascular and respiratory test” section below, 7-day averages for weather and air pollution data have been used and holiday periods and bank holidays have been omitted from the sample.

From a preliminary data correlation analysis, it was evident, that at least for some ICD-10 categories (mainly ICD-10 = J, respiratory diseases), a strong correlation between temperature (Chan et al. 2013) and hospitalizations was present. To account for this fact, we normalized the data set also by temperature.

No further significant correlations with other meteorological values—windspeed, precipitations, pressure, and humidity—has been found (Zhang et al. 2014), and therefore, no further normalizations have been added to the dataset.

### Correlation analysis and DLNM

On such normalized data set, the hypothesized association between air pollution and number of hospitalizations was analyzed using at first a simple correlation analysis. As the association between air pollution and respiratory illness may be delayed in time (Zhang et al. 2018; Sinclair and Tolsma 2004), a potential lag effect from 0 to 10 days has been taken into consideration (Taj et al. 2017; Lall et al. 2011). To further explore the lag effect, on the data that showed bigger potential association, the correlation analyses was combined with a distributed lag nonlinear model (DLNM) (Gasparrini et al. 2010, 2012). The lag cumulative effect was considered over all lags from 0 to 10 days. The chosen method was the Almon method (Almon 1965), which can handle DLNM (Almon lag model 2018), is largely used, and for which several open softwares are available. The distributed lag analysis has been performed in Statistica software using the Almon lag model (Statistica 2018).

The model can be shortly written as

$$ y(t)=\sum \limits_{i=\phi}^k{\beta}_{{}_ix\left(t-i\right)+\varepsilon (t)} $$

where the *x*_*i*_ predictor variables of *y* used in the model represent observations made periodically during a continuous time period beginning at some time before *y* was observed and ending at the time of observation of *y*. Models of this kind are known as distributed lag models and are useful when changes in the independent variable *x* have an effect on the value of *y* over many samples of *y*. Typically, in this bivariate distributed lag model, if *x* and *y* are observed at identical periods at the same frequency, *t*, bivariate observations will be made of *y*(*t*) and *x*(*t*). The percentage of number of patients’ increase was calculated based on the results of multiple regression, where response variable was number of patients (normalized by the day) and independent variables were pollution level and temperature. The increase of number of patients was estimated using coefficient of regression (slope) for pollution level multiplied by 10 (number of units of pollutants).

### Cardiovascular and respiratory test

A subset of data has been selected (cardiovascular diseases and respiratory diseases—ICD-10: I10–I15, I20–I24, I26, I40, I41, I44–I49, I50, I60–I68, I74, I80–I82, J00–J46) to focus the analysis on both a broader dataset first, and narrowing data next with a higher probability of association, as well as to test the sensitivity of the results. The pollutant (PM_2.5_ and PM_10_) levels and data on weather conditions were computed as a 7-day moving average. Furthermore, data points of Saturday, Sunday, and holidays were omitted. Regression analysis was further performed using the following variables, separately for each city:

- Logarithm values from the average of the last 7 days for particulate matter concentrations PM_10_ and PM_2.5_—where PM logarithm: *y* = (/100)% *x*)
- Average of the last 7 days for weather data (temperature—n °C), maximum wind speed (in 10 m/s), humidity (in %), pressure (in hPa), and sum of precipitation (in 10 mm)
- The values of average squares from the last 7 days for weather variables
- Zero variables for days of the week

## Results

### Descriptive statistics of the study setting

The hospitalizations statistics per ICD category are displayed in Table 1.

**Table 1:** Mean hospitalizations per day per ICD-10

Mean visits per day	Hospitalization diagnosis (ICD-10)															
	F (mean)	SD	G (mean)	SD	H (mean)	SD	I (mean)	SD	J (mean)	SD	L (mean)	SD	S (mean)	SD	T (mean)	SD
Białystok	315.8	216.9	68.7	42.6	50.8	29.9	548.9	367.9	940.7	687.7	66.3	43.2	207.4	105.6	3.6	2.3
Bielsko-Biała	143.5	98.4	38.0	25.1	24.3	13.9	345.0	231.1	499.8	380.4	47.3	31.3	114.4	56.2	3.3	1.29
Gdańsk	456.2	296.3	100.0	65.9	59.0	28.1	953.0	636.1	1282.0	862.1	92.5	59.7	278.7	137.6	3.6	2.16
Kraków	733.7	495.8	163.2	110.8	87.5	48.6	1432.5	969.4	2160.4	1491.0	130.6	83.4	389.0	182.7	0.0	0.0
Warszawa	1852.8	986.6	269.3	169.3	230.1	127.9	3517.8	2335.9	3539.3	2317.4	239.7	148.1	1034.8	480.8	17.2	7.4

A proportionally large variability due to seasonality and day of the week was clearly observed (large SDs).

The air pollutant statistics are displayed in Table 2. The cities that displayed the highest pollution index were Krakow (mean NO_2_ 65.34 ppb, PM_2.5_ 68.38 μg/m^3^, PM_10_ 89.85 μg/m^3^) and Warsaw (mean NO_2_ 61.82 ppb, PM_2.5_ 38.09 μg/m^3^, PM_10_ 57.15 μg/m^3^). For several cities, the air pollutant values have often crossed significantly the EU guideline “Air Quality Standards” level, and in some cases (particulate matter and NO_2_), the mean value during the study period was already above such limits (Air Quality Standards 2008; Dąbrowiecki et al. 2018).

**Table 2:** Descriptive statistics of air pollution

	Gdańsk^a^		Białystok^a^		Bielsko-Biała^a^		Kraków^b^		Warszawa^c^		AQS
Variable	Mean	Data range (from-to)	Mean	Data range (from-to)	Mean	Data range (from-to)	Mean	Data range (from-to)	Mean	Data range (from-to)	
NO (ppb)	21.61	0.82–223.73	9.48	0.1–173.01	22.08	0–325.47	365.42	20.54–906.76	67.73	3.52–401.22	
NO_x_ (ppb)	74.78	6.54–655.63	36.58	1.39–314.2	73.12	0–611.14	247.86	20.00–1506.31	158.04	16.23–759.58	
NO_2_ (ppb)	32.94	4.72–102.78	24.98	2.01–93.6	42.40	0–141.02	65.34	13.00–160.15	61.82	9.57–177.68	50
O_3_ (ppb)	66.90	3.6–146.28	73.68	0–174	76.28	0–180.92	68.35	0.00–187.74	67.03	1.83–178.94	120
SO_2_ (ppb)	10.68	1.4–420.99	6.35	0.97–58.14	17.69	0–140.19	9.97	1.31–68.00	13.00	0.98–118.40	125
PM_2.5_ (mg/m^3^)	24.35	0–126	36.64	0–261	0.00	0–0	68.38	10.55–448.12	38.09	5.45–165.58	25
PM_10_ (mg/m^3^)	63.65	3.24–859.25	54.01	0–402.6	76.65	0–462.37	89.85	14.82–522.92	57.15	9.92–277.56	40
PM_10__24 (mg/m^3^)	26.60	0–145	21.82	0–129.7	37.09	0–319.7	33.86	0.00–279.30	23.23	4.54–128.10	25
PM_2.5__24 (mg/m^3^)	14.41	0–104	20.14	0–134.17	29.80	0–280.3	47.64	0.00–333.80	33.22	0.00–151.80	40

*AQS* Air Quality Standards for the EU

^a^One thousand two hundred fifty-five measure days (except PM2.5_24 and PM10_24 1216 days)

^b^One thousand twelve measure days (except PM2.5_24 and PM10_24 990 days)

^c^One thousand forty-seven measure days (except PM2.5_24 and PM10_24 1016 days)

**Fig. 1:**
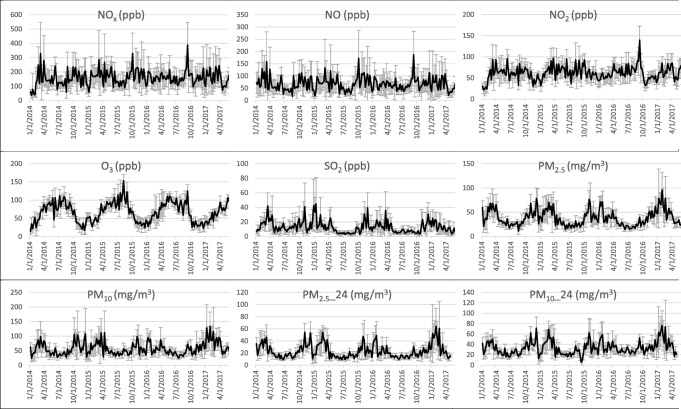
Mean weekly pollutant concentrations in Warsaw. SDs are presented as error bars

**Fig. 2:**
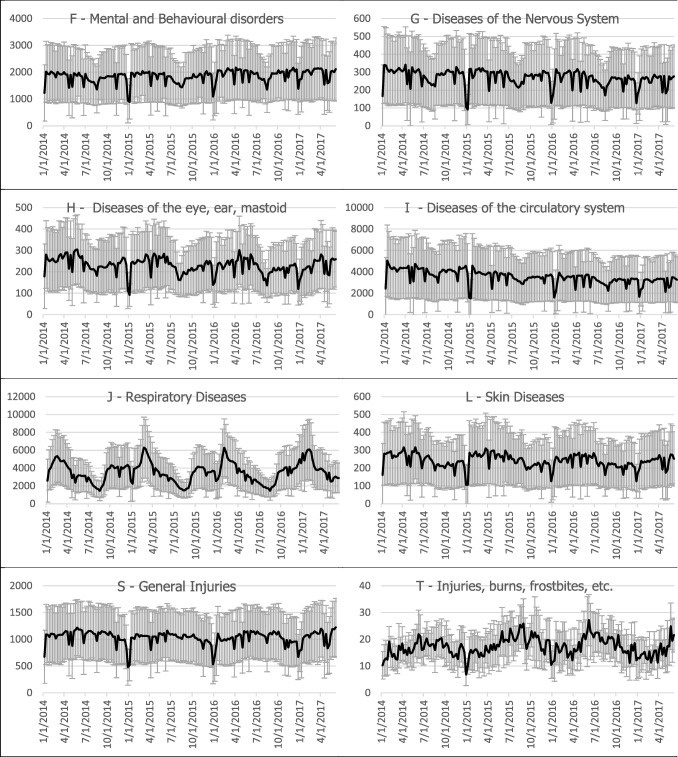
Mean number of patients per day in Warsaw based on weekly data for various types of ICD-10 categories. SDs are presented as error bars

In Figs. 1 and 2, the hospitalization statistics and the air pollution values are graphically displayed for the largest city, Warsaw. It has been chosen as a representative city because of the highest number of inhabitants and highest pollution grade.

The meteorological statistics are displayed in Table 3. The values are coherent with a continental climate region with relatively little wind—in comparison with other climates, e.g., Mediterranean—so with a limited chance for the weather to dilute the air pollutants.

**Table 3:** Meteorological values statistics

	Daily data	Mean	SD	Min	P25	Median	P75
Białystok	Temperature °C	7.7	8.2	− 17.9	1.8	6.8	14.4
	Main wind speed (km/h)	9	3.4	2	6.7	8.3	10.6
	Precipitation (mm/month)	65.3	394.4	0	0	0	2
Bielsko-Biała	Temperature °C	9.4	7.8	− 18.1	3.6	9.2	15.5
	Main wind speed (km/h)	9.6	5.6	2.4	5.9	7.6	11.3
	Precipitation (mm/month)	51.5	347.9	0	0	0	2.8
Gdańsk	Temperature °C	8.9	6.7	− 9.6	3.8	7.9	14.8
	Main wind speed (km/h)	14.2	5.3	3.9	10.6	13.1	17
	Precipitation (mm/month)	46.2	333.5	0	0	0	1.5
Kraków	Temperature °C	9.2	8	− 19.8	3.4	8.5	15.6
	Main wind speed (km/h)	10.8	5.9	0.9	6.7	9.4	13.5
	Precipitation (mm/month)	52.8	355	0	0	0	1.8
Warszawa	Temperature °C	9.5	8.4	− 15.6	3.6	8.5	16.2
	Main wind speed (km/h)	13	5.2	3.7	9.3	12.2	15.9
	Precipitation (mm/month)	54.7	361.9	0	0	0	1.5

### Relationship of meteorological values with hospitalizations

To evaluate the relationships between weather variables and the number of hospitalized patients, we calculated the corresponding correlation coefficients. Through this, we evaluated the strength of relationships and the direction (negative or positive) of their influence. Squared values of correlation coefficients (*R*^2^) in case of temperature were recorded between 35 and 50% (depending on the ICD-10 category), for wind speed 1–3% and for precipitation only about 1%. As an example, the correlation coefficients for all the measured meteorological variables for ICD-1 = J (i.e., respiratory diseases) are reported in Table 4.

**Table 4:** Correlation coefficients between weather variables and number of patients ICD-10 = J (normalized by day of the week)

City	Temperature °C	Wind speed km/h	Precipitation (mm)
Białystok	− 0.6247	0.1724	0.0589
	*p* = 0.000	*p* = .000	*p* = 0.037
Bielsko-Biała	− 0.6010	0.1790	− 0.0625
	*p* = 0.000	*p* = 0.000	*p* = 0.027
Gdańsk	− 0.7198	0.1134	− 0.0950
	*p* = 0.000	*p* = 0.000	*p* = 0.001
Kraków	− 0.7124	0.1044	− 0.0229
	*p* = 0.000	*p* = 0.000	*p* = 0.418
Warszawa	− 0.7114	0.1102	0.1195
	*p* = 0.000	*p* = 0.000	*p* = 0.000

Relationship of different pollutants and effects on hospitalizations

For each pollutant and for each city, a correlation table was generated plotting the ICD-10 hospitalization diagnosis versus the lag (days from 0 to 10). The highest absolute value of the correlation coefficients recorded by this methodology is reported in Table 5: the highlighted values represent the highest 25th percentile. The highest recorded correlation is clearly identified in the ICD-10 J column (i.e., respiratory diseases).

**Table 5:** Correlation coefficients between pollutants and patients hospitalized in the different ICD-10 categories

		Hospitalization ICD10 categories							
Pollutant	City	F	G	H	I	J	L	S	T
NO_*x*_	Krakow	0.039	0.067	0.058	0.078*	0.191**	0.071	0.071	0.000
	Gdansk	0.032	0.076*	0.097*	0.109*	0.115*	0.076*	0.040	0.040
	Bielsko Biala	0.046	0.032	0.058	0.057	0.176**	0.054	0.081*	0.070
	Bialystok	0.028	0.087*	0.066	0.044	0.158**	0.068	0.063	0.052
	Warsaw	0.085*	0.092*	0.038	0.037	0.180**	0.079*	0.093*	0.058
NO	Krakow	0.057	0.085*	0.032	0.076*	0.221**	0.053	0.084*	0.000
	Gdansk	0.057	0.054	0.085*	0.096*	0.147*	0.093*	0.066	0.063
	Bielsko Biala	0.042	0.029	0.077*	0.025	0.145*	0.053	0.082*	0.063
	Bialystok	0.023	0.098*	0.042	0.054	0.120*	0.045	0.065	0.048
	Warsaw	0.069	0.112*	0.036	0.054	0.170**	0.060	0.095*	0.057
NO_2_	Krakow	0.041	0.054	0.071	0.108*	0.228**	0.072	0.155**	0.000
	Bielsko Biala	0.033	0.052	0.047	0.053	0.232**	0.060	0.118*	0.052
	Bialystok	0.044	0.081*	0.064	0.089*	0.148*	0.055	0.060	0.074*
	Warsaw	0.076*	0.076*	0.045	0.031	0.129*	0.067	0.135*	0.066
SO_2_	Krakow	0.024	0.028	0.068	0.109*	0.202**	0.094	0.126*	0.000
	Gdansk	0.050	0.045	0.093*	0.053	0.033	0.066	0.082*	0.063
	Bielsko Biala	0.053	0.039	0.048	0.078*	0.228**	0.073*	0.080*	0.056
	Bialystok	0.026	0.044	0.033	0.085*	0.208**	0.047	0.058	0.055
	Warsaw	0.065	0.104	0.075*	0.110*	0.168**	0.078*	0.065	0.082*
PM_2.5_	Krakow	0.035	0.025	0.065	0.062	0.175**	0.060	0.095*	0.000
	Gdansk	0.080*	0.054	0.047	0.099*	0.220**	0.070	0.074*	0.042
	Bialystok	0.042	0.053	0.061	0.081*	0.245**	0.051	0.060	0.048
	Warsaw	0.062	0.067	0.060	0.042	0.279**	0.047	0.084*	0.098*
PM_10_	Krakow	0.042	0.026	0.065	0.033	0.209**	0.051	0.085*	0.000
	Gdansk	0.079*	0.076*	0.099	0.116	0.055	0.044	0.058	0.041
	Bielsko Biala	0.042	0.047	0.072	0.041	0.214**	0.067	0.109*	0.050
	Bialystok	0.045	0.045	0.075*	0.074*	0.178**	0.040	0.062	0.048
	Warsaw	0.082	0.054	0.049	0.021	0.233**	0.062	0.106*	0.092*
PM_2.5_ 24 h	Krakow	0.038	0.036	0.047	0.047	0.163**	0.048	0.081*	0.000
	Gdansk	0.050	0.084*	0.047	0.053	0.185**	0.083*	0.049	0.043
	Bielsko Biala	0.067	0.039	0.081*	0.046	0.162**	0.054	0.111*	0.070
	Bialystok	0.016	0.066	0.050	0.071	0.248**	0.020	0.101*	0.053
	Warsaw	0.025	0.064	0.061	0.033	0.276**	0.049	0.102*	0.079*
PM_10_ 24 h	Krakow	0.034	0.027	0.054	0.024	0.189**	0.045	0.101*	0.000
	Gdansk	0.063	0.106*	0.040	0.077*	0.202**	0.085*	0.038	0.033
	Bielsko Biala	0.051	0.019	0.070	0.042	0.160**	0.051	0.120*	0.058
	Bialystok	0.080*	0.075*	0.038	0.097*	0.166**	0.034	0.060	0.031
	Warsaw	0.081*	0.022	0.055	0.036	0.265**	0.049	0.119*	0.071
O_3_	Bielsko Biala	0.075*	0.066	0.080*	0.059	0.145*	0.087*	0.051	0.044
	Bialystok	0.038	0.045	0.056	0.047	0.130*	0.108*	0.080*	0.058
	Warsaw	0.031	0.016	0.105*	0.019	0.139*	0.169**	0.131*	0.052
25th percentile		0.035	0.039	0.047	0.042	0.148	0.049	0.063	0.040

*Significant correlation at 0.01 significance level; **Highlighted values represent the strongest (and significant) correlation coefficient > 0.15

### Respiratory disease hospitalizations

The deeper analysis using the Almon model algorithm has been applied to the respiratory disease sub-data, and the *P* values for the distributed lag model (Almond method) were plotted, where the pollutant was the independent variable (cause) and the number of events (hospitalizations) was the dependent variable (always with lags from 0 to 10 days). The results of the highest recorded correlation/lag day, as well as the calculated coefficient of % increase per each 10 units of increased pollutant at the specific identified lag are displayed in Table 6.

**Table 6:** Percent increase of hospital admissions for respiratory disease/lag (days)

	Gdańsk		Białystok		Bielsko-Biała		Kraków		Warszawa		All cities^a^
Variable	%	Lag (days)	%	Lag (days)	%	Lag (days)	%	Lag (days)	%	Lag (days)	%
NO (ppb)	1.0	6	1.9	4	1.4	6	0.2	2	0.8	6	0.3
NO_*x*_ (ppb)	0.30%	3	1.3	4	0.7	5–6	0.3	2	0.5	5–6	0.4
NO_2_ (ppb)	A		2.9*	4	3.5	4	2.6	1–2	1.3*	4	2.4
O_3_ (ppb)	A		− 1.7*	9	− 2.1*	10	A		− 1.6*	9	− 1.2
SO_2_ (ppb)	0.3*	3	12.7	0	5.4	5–7	7.5*	3	3	8	1.6
PM_2.5_ (mg/m^3^)	3.1	5	2.4	5–6	A		0.8*	2	3.4	7	1.3
PM_10_ (mg/m^3^)	0.1*	3	1	5	1.1	6	0.9	3	1.6	7	0.6
PM_10__24 (mg/m^3^)	3.1	7	2.8	5–6	1.7	5–6	1.4	3	3.5	7	1.9
PM_2.5__24 (mg/m^3^)	3.6	7	4.5	6–7	1.9	5	1.4	4	4.5	7	2.3

All *p* values are below 0.000 with exception Gdańsk values of SO_2_ (*p* value 0,237) and PM_10_ (*p* value 0.054)

*%* % increase of hospitalizations per each 10 additional pollutant units, *Lag days* intersection of the lowest P value from Almon model and the strongest correlation coefficient, *A* measurements not available

*Highlighted values represent a lower correlation coefficient (as seen in Table 5)

^a^The results for all cities together were calculated using multiple linear regression were city was treated as dummy variable

Several pollutants show a statistically significant and correlated increase in hospitalizations, with the largest effect (as well as consistent among the different cities) being the one of the particulate matter, PM_2.5_ and PM_10_.

### Subset analysis on cardiovascular disease and respiratory disease hospitalizations

**Fig. 3:**
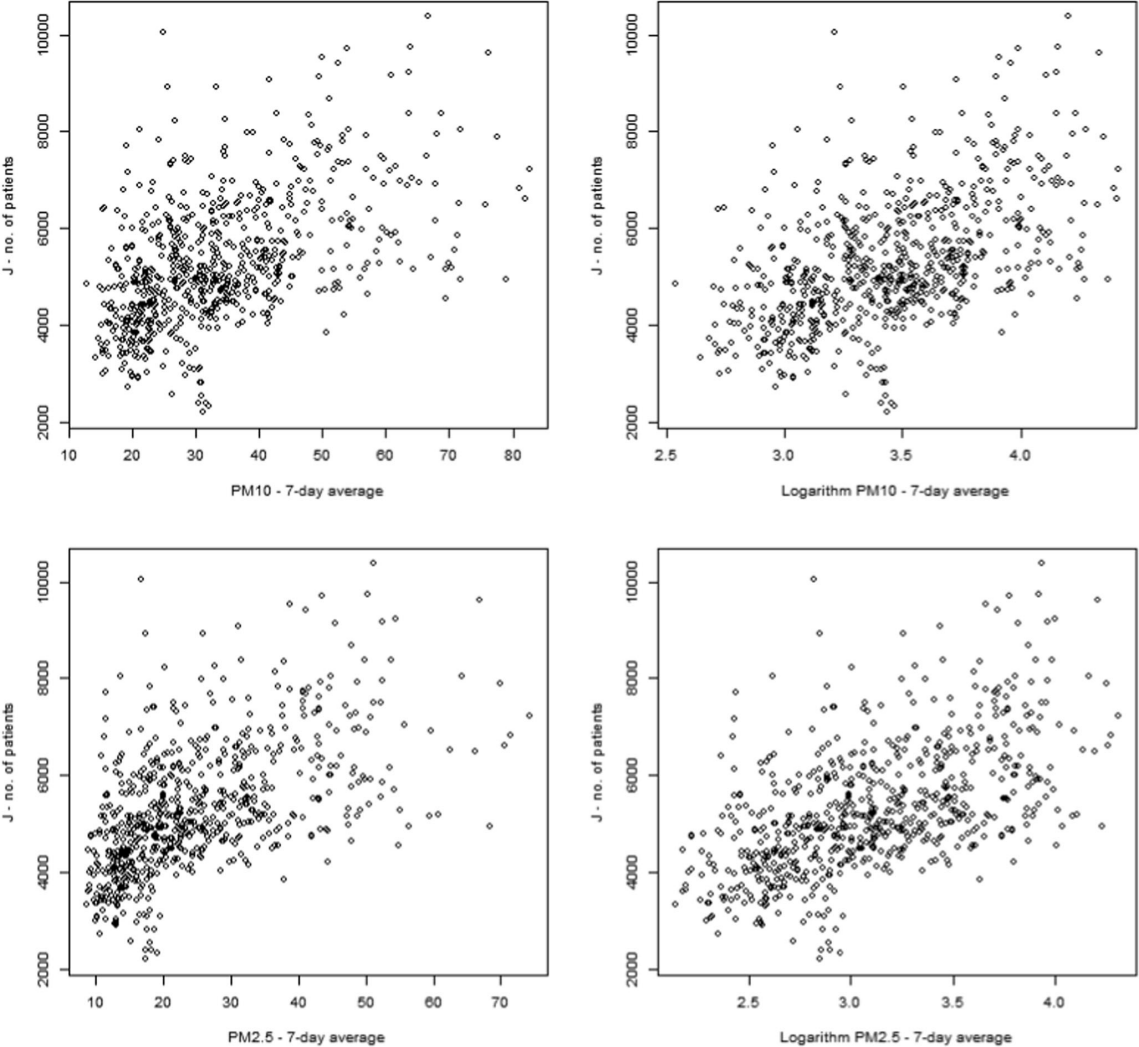
Relationships between the number of patients with respiratory diseases (J) and PM particulate matter concentration level in Warsaw (logarithm of 7-day average)

The results of the subset data analysis on cardiovascular disease and respiratory tract disease with the 7-day average pollutant values and the method described in “Cardiovascular and respiratory test” section have provided a similar result, displayed in Table 7. In Fig. 3, the sample plot of the respiratory patients in Warsaw (ICD-10=J) versus the 7 day particulate matter concentration average (linear and logarithm) is being displayed.

**Table 7:** Percent increase in patients per each 10-unit increase in pollutant concentration

		Białystok		Bielsko-Biała		Gdańsk		Kraków		Warszawa		All cities^a,b^	
	Pollutant	%	*N*	%	*N*	%	*N*	%	*N*	%	*N*	%^a^	*N*^b^
Respiratory	PM10	1.70%	25	2.30%	18.3	1.40%	26.8	1.40%	46.3	2.10%	111.7	1.78%	228.1
Disease	PM2.5	1.60%	23.2	1.27%	9.9	0.94%	17.6	1.08%	35.3	2.00%	106	1.51%	192
Cardiovascular	PM10	0.70%	5.7	0.70%	3.5	0.50%	7.3	0.50%	9.7	0.01%	0.3	0.27%	26.5
Disease	PM2.5	0.90%	6.9	0.40%	1.9	0.20%	3	0.10%	2.9	0.44%	22.9	0.37%	37.6

*%* increase of the percent of patients/day per each 10 units of increase in pollutant concentration, *N* number of additional patients increase per day per each 10 units of pollutant concentration increase

^a^Increase of the patients per day for all cities was calculated as weighted average where the weight was the number of patients per total study period

^b^Number of additional patients increase per day for all cities was calculated as the sum of all increases/day

For all examined cities, the impact of changes in the average PM_10_ concentration level from the last 7 days on hospital admissions due to respiratory diseases is statistically significant at the significance level of 0.01. With 10% increase in PM_10_ concentration from the mean, for example, the number of patients increases on average by 25 patients (1.7% of the average number patients) in Bialystok, 26.8 (1.4% of mean) in Gdansk, 46 (1.4%) in Krakow, 18 patients (2.3% of average) in Bielsko-Biała, and 111.7 people (2.1%) in Warsaw. The average PM_10_ concentration from the previous 7 days had a statistically significant impact on hospital admissions due to cardiovascular system diseases for all cities studied except of Warsaw. Although statistically significant, correlations were weak. Similar dependencies apply to the model for PM_2.5_ and cardiovascular disease. The biggest effect of the increase in concentration particulate matter by 10% is an increase of 0.9% in the number of patients in Bialystok

## Discussion

### Key results

In this analysis, we have found positive associations between ambient levels of pollutants (mainly PM_2.5_ and PM_10_) and hospitalizations. A positive association between air pollution and acute respiratory disease health impact/hospitalization was to be expected (Zhang et al. 2018; Sinclair and Tolsma 2004, Sinclair et al. 2010; Vahedian et al. 2017). The pollution levels at which these results were recorded, even though on a proportionally high pollution range (Air Quality Standards; Directive 2008; WHO Air Quality 2005; WHO 2013), were still not in a “critical” range similar to the London smog 1952 or the Asian smoke-haze event of 1997 (Bell and Davis 2001and Bell et al. 2004, Heil and Goldammer 2001) (see Table 2).

The peak effect on hospitalizations increase has been found with a time lag of 3–6 (sometimes up to 7) days (see Table 6). Such lag effect for respiratory disease hospitalizations show similarities with earlier findings (Sinclair and Tolsma 2004; Sinclair et al. 2010; de Souza et al. 2014).

Several mechanisms have been suggested (Esposito,  Tenconi et al., 2014) to explain the adverse effects of air pollutants. The most consistent and most widely accepted explanation (Chauhan and Johnston 2003; Arbex et al. 2012) is that, once in contact with the respiratory epithelium, high concentrations of oxidants and pro-oxidants in environmental pollutants such as PM of various sizes and compositions and in gases cause the formation of oxygen and nitrogen-free radicals, which in turn induce oxidative stress in the airways. In other words, an increase in free radicals that are not neutralized by antioxidant defenses initiate an inflammatory response with release of inflammatory cells and mediators (cytokines, chemokines, and adhesion molecules) that reach the systemic circulation, leading to subclinical inflammation, which not only has a negative effect on the respiratory system but also causes systemic effects. These processes may take a discrete amount of days to lead to clinically relevant symptoms that require, due to their severity, medical attention and/or hospitalization.

A more limited correlated and statistically significant association has been found between the different pollutant levels and cardiovascular disease (CVD). Several years ago, in a study covering some Eastern European cities, on a smaller sample case (Katsouyanni et al. 1997) and looking at mortality rate, and not on hospitalization rates, a somehow similarly trending result has been reported (Samoli et al. 2001).

To some extent, the result of poorer correlation with CVD could be explained first in a high preexisting baseline, i.e., the underlying relatively high prevalence of cardiovascular disease which shows a relatively high baseline rate of CVD hospitalizations (Szafraniec-Burylo et al. 2016), rendering the peaks which might be generated by the excess exposure to pollutants less visible. Another possible input in the study results is the source of the pollutant. In general, the “smoke” pollution in Poland is particularly influenced by a high degree of utilization of charcoal as heating (Nabrdalic and Samora 2018). In all the European Union, 80% of private homes using coal are in Poland. Scientific debate is currently ongoing (Hime et al. 2018) on the health effects on particulate pollution depending on the source of pollutant. The charcoal burning fumes would contain PM particles with a higher SO_*x*_-bound component than, for example, diesel exhaust and might therefore have a greater influence on the respiratory tract (sulfur oxides are toxic urticants). In addition, other bias factors such as underlying morbidity, age, etc., could have had an impact to the results.

### Study limitations

Limitation of this study are represented by the missing stratification of the hospitalizations between age groups and gender, which could help identifying clearer trends and additional subanalysis could have been made. In addition, as the data source consisted of aggregated daily records of hospitalizations, the statistical significance of the results is weaker. As a last point, while the temperature effect on hospitalization (e.g., flu season related hospitalizations) has been tackled normalizing the sample data (as described in the “Sample preparation” section), such normalization does not totally separate the potential cause/effect or combined effect of pollution/season from the results.

### Generalizability and further analysis

The overall number of hospitalizations captured by the analysis (over 20 million) is large compared to literature (Moore et al. 2016; Zhang et al. 2018; Stieb et al. 2009), in particular in Europe, and the results per se could therefore be interesting to also help predict hospitalization trends and quantify the needs for environmental preventive measures to help minimize the healthcare impact and costs associated with such events. It is important to note, however, that the aggregated daily records’ data source do constitute a limitation to the strength of the statistical analysis.

Also for this reason, on the same dataset, further analysis could be performed using different statistical methodologies, e.g., a case-crossover data setup (Lu and Zeger 2007) or artificial neural networks (Fang 2018; Polezer et al. 2018) to further test the sensitivity of these results.

## Conclusions

Ambient air pollution exposure increases were associated with an increase of hospitalizations due to respiratory tract diseases in a large time-series observation in five major polish cities in the years 2014–2017. The most prominent effect was recorded with the correlation of PM_2.5_ and PM_10_. There was weak evidence of short-term associations between peaks of air pollution concentrations and increased hospitalizations for cardiovascular diseases. A further work on a dataset enabling a better stratification of the sample (e.g., age, gender, and likewise a detailed admission analysis, e.g., sub-stratum for COPD, lower respiratory tract infection, asthma, etc.) would provide a better insight on the subject matter.

## Abbreviations

COPD: Chronic obstructive pulmonary disease
DLNM: Distributed lag nonlinear model
ICD: International Classification of Diseases
NHF: National Healthcare Fund
PM: Particulate matter
PM_2,5_: Particulate matter 2.5 μm or less in diameter, generally described as fine particles
PM10: Particulate matter 10 μm or less in diameter
SD: Standard deviation
